# A Qualitative Study on Children’s Digital Media Use and Parents’ Self-interest

**DOI:** 10.1007/s10826-021-02074-3

**Published:** 2021-09-23

**Authors:** Suzanne M. Geurts, Ina M. Koning, Helen Vossen, Regina J.J.M. Van den Eijnden

**Affiliations:** 1 Interdisciplinary Social Science, Youth Studies, Utrecht University, Padualaan 14, 3584 CH Utrecht, The Netherlands; 2 Education and Pedagogy, Clinical Child and Family Studies, Utrecht University, Heidelberglaan 1, 3584 CS Utrecht, The Netherlands

**Keywords:** Children’s digital media use, Parents’ self-interest, Qualitative research

## Abstract

This qualitative study provides insight into the role of parents’ self-interest in digital media use of children in different age groups. We conducted 31 semi-structured interviews with fathers/mothers of children aged 3–16 years who were recruited via targeted sampling. A deductive and inductive content analysis was applied. Results show that parents’ self-interest in letting children use digital media includes being able to do other tasks without being bothered, having some me-time, managing children’s behavior, avoiding discussions, having moments to use digital media themselves and spending quality-time together. In addition, we found that the manner in which parents let children use digital media out of self-interest seems to depend on age. With younger children, parents initiate digital media use or set times at which children are allowed to use digital media. With older children, parents use a passive manner by omitting restrictive responses to their children’s media use. Current findings can be used to inform interventions aimed at reducing children’s screen time.

## Highlights

- Parents’ have a self-interest in their kids’ digital media use.
- Parents of younger children use digital media out of self-interest in a more active way.
- Parents of older children use digital media out of self-interest in a more passive way.
- The possibility to do other tasks undisturbed is the most important reason to let their children use digital media.

Present days, digital devices such as smartphones, laptops, and tablets have become essential to the lives of children and youth. In the Netherlands, almost all youth between the ages 12 and 15 have access to a smartphone (98.7%) and a laptop (91.7%), 78.1% have access to a tablet at home and 58.5% to a game console (CBS, 2019). In addition, children start using digital media at an increasingly younger age. For instance, research shows that in 1970 children started watching television regularly at the age of four, whereas nowadays children are four months old when they use digital devices (Chassiakos et al., 2016). Youth uses digital devices primarily for social media (e.g. Snapchat, Whatsapp and YouTube) and to play online games. The use of such digital media has many advantages including being able to easily maintain contact with friends, easy access to information and the opportunity to learn playfully and interactively (Chassiakos et al., 2016; Kervin, 2016; Siddiqui & Singh, 2016). Despite these advantages, digital media use can have negative consequences for youth’s wellbeing. For example, exposure to violent media can contribute to the development of anxiety, nightmares and aggressive behavior by children (American Academy of Pediatrics, 2009). Intensive use of digital media has regularly been associated with sleeping problems (Cain & Gradisar, 2010; Hale & Guan, 2015; LeBourgeois et al., 2017), poor school performance (Luo et al., 2020; Sharif et al. 2010; Van den Eijnden et al., 2018), eye complaints (Choi et al., 2018; Kim et al., 2016), unhealthy eating behavior (Pearson & Biddle, 2011) and a higher BMI (Body Mass Index; Tremblay et al., 2011) among youth. Furthermore, intensive use of social media and gaming can result in problematic, addiction-like use, meaning that children and youngsters are no longer able to control their online behavior (Griffiths et al., 2014; Müller et al., 2016; Van den Eijnden et al., 2016). To avoid possible negative effects of digital media use on important health-related behaviors such as exercise, sleeping, face-to-face interaction and imaginary play (Yogman et al., 2018), a balance between screen and non-screen activities is essential (Subrahmanyam et al., 2000). Parents play an important role in helping their children find this balance (Meeus et al., 2018), as they are the ones providing or restricting access to digital devices and limiting their children’s digital media use by setting rules (or not) (Livingstone et al., 2015).

Based on previous research on substance use, several existing parenting strategies were measured and related to digital media, though, without any success. For example, contrary to the protective influence of parental rules about alcohol on the alcohol use of adolescents (Koning et al., 2014; 2015), rules regarding internet use were not or only weakly related to adolescents’ social media and game use (e.g. Koning et al., 2018). This led us to identify major differences between previously studied risk behaviors (e.g. alcohol use) and digital media use, such as 24/7 availability, no age limits, as well as the benefits parents may experience and its subsequent encouragement of the use of digital media (we refer to the latter as parents’ self-interest in children’s digital media use). Since digital media are very useful in keeping children entertained, children’s media use can have benefits for parents. For example, having their child watch a movie or play a game on a tablet allows parents to do household chores, or to have some downtime (Beyens & Eggermont, 2014). In a study conducted by He et al. (2010), 34.4% of parents of 10-11-year olds indicated that they use the television and/or computer to entertain their children. Moreover, a more recent Dutch study (Nikken, 2019) demonstrated that during previous years parents have developed more positive attitudes towards the use of digital media as a babysitter, as a way to create time for themselves, and as a reward for proper eating behavior or as a helpful tool when putting children to bed. The benefit parents achieve from children’s media use may particularly be the case in times of COVID-19. Restrictions aimed at decreasing the spread of COVID-19 (such as the closure of, amongst others, schools and sports clubs and the strong advice to work from home) result in children and parents spending more time together at home. In light of these circumstance, it may even be more beneficial for parents to let their children use digital devices to have time for their own tasks. The benefits for parents associated with children’s digital media use might form an obstacle in limiting children’s screen time (Evans et al., 2011). Research shows that when parents let their children use digital media for reasons of self-interest; i.e. to have some downtime or to finish work, children likely spend more time behind a screen (Beyens & Eggermont, 2014; Hawi & Rupert, 2015). Thus, if parents’ self-interest is increasingly used as a motive for allowing children to use digital media, this might pose an increased risk to the healthy development of youth. Supported by several scholars that urge to move beyond simple cause-effect relationships (e.g. Blum-Ross & Livingstone, 2020; Linebarger et al., 2017), a qualitative design may contribute to a better understanding of the role of parents’ self-interest. Therefore, the present study investigates how parents deal with their children’s digital media use out of self-interest (i.e. to elicit personal benefit) and which types of self-interests play a role herein.

## Children’s Digital Media Use and Parents’ Self-interest

Some qualitative research has been conducted on parents’ reasonings for allowing children to watch television. These studies show that parents do so, among other things, as a family activity, as part of the daily routine (for example in the evening before going to bed; Götz et al, 2007; He et al., 2005) or to promote communication between parent and child (Evans et al., 2011). It’s also known that parents use television as a tool to regulate children’s behavior (Evans et al., 2011; He et al., 2005; Hesketh et al., 2012), to distract children from pain, to positively influence children’s mood, or to offer them a good alternative to enjoy themselves during inclement weather or illness (Götz et al., 2007). In addition, studies show that having a moment to relax, having time to finish other tasks (Bentley et al., 2016; Evans et al., 2011; Götz et al., 2007; He et al., 2005; Hesketh et al., 2012) or avoiding conflicts between parent and child and quarreling between siblings are motives of parents for letting children watch television (Evans et al., 2011; He et al., 2005; Hesketh et al., 2012). An observational study by Radesky and colleagues (2014) on the use of smartphones by parents and children in fast-food restaurants showed that mobile devices, just like televisions, offer a solution for parents to regulate children’s behavior. Together, these findings illustrate that children’s digital media use is not only motivated by the needs of children themselves, but also by the needs of their parents.

Although these studies provide information about parents’ benefits associated with children’s digital media use, previous research focused mainly on parents of children under the age of 6 (Beyens & Eggermont, 2014; Götz et al., 2007; He et al., 2005; Hesketh et al., 2012; Nikken, 2019). Research on this topic among parents of adolescents is scarce. As a result, it is not known whether parents’ self-interest also plays a role concerning adolescents’ digital media use and, if so, whether the same types of parents’ self-interest are applicable in younger children’s media use. Furthermore, nearly all previous studies concerned screen use include television viewing, playing video games and using the computer (Beyens & Eggermont, 2014; Evans et al., 2011; Götz et al., 2007; He et al., 2005; Hesketh et al., 2012), and not the use of modern digital technologies, such as laptops, smartphones and tablets (Bentley et al., 2016). Unlike televisions, game consoles and computers, these portable digital devices can be used anywhere at any time. This makes it plausible that parents’ self-interest will be expressed in additional forms. Moreover, there is no current insight into how parents’ self-interest plays a role in dealing with children’s digital media use. For instance, do parents initiate digital media use among their children to satisfy their self-interest? Do parents, out of self-interest, allow digital media use when children ask for it? Or do parents omit restrictive responses to their children’s digital media use for reasons of self-interest? It is imperative to increase our understanding and awareness of how parents consciously or unconsciously create or utilize moments during which their children use digital media and which forms of self-interest play a role in this. This can help parents to find or maintain the right balance for their children between activities with and without a screen. Therefore, more research into how parents benefit from the digital media use of children in different age categories, and what these benefits exactly are, is desirable.

To fill the gaps in the literature concerning the role of parents’ self-interest in children’s digital media use, this qualitative study aims to answer the following research questions: 1) In which ways do parents deal with digital media use of children aged 3 to 16 years out of self-interest? 2) Which motives do parents have for their self-interest in allowing children to use digital media? 3) To what extent do the different motives of parents’ self-interest provoke different ways of dealing with children’s media use? 4) To what extent do the answers to these questions differ for parents with children in different age categories? Based on previous knowledge on parents’ self-interest regarding children’s use of more traditional media (e.g. Evans et al., 2011), parental mediation (Koning et al., 2018), as well as our knowledge about the role of parents in alcohol use and studies that, for example, demonstrated that parents provide alcohol to their child themselves (e.g., Van der Vorst & Engels, 2008), we expect that there are three ways in which parents deal with their children’s media use out of self-interest, namely by initiating digital media use, by omitting restrictive responses and by allowing digital media use when asked. This article is a revised and translated version of the previously published Dutch article (it shares the same research questions, data, results and discussion; Geurts et al., 2020).

## Methods

### Design

In order to obtain new and detailed information about how parents deal with their children’s digital media use out of self-interest and which forms of self-interest play a role in this, a qualitative method is applied in this study (Rich & Ginsburg, 1999).

### Participants and Recruitment

Participants were recruited by targeted sampling through an invitation on Facebook and from the personal networks of the research assistants who conducted the interviews. The inclusion criterion was ‘Dutch-speaking parents having at least one child aged 3 to 16 years. In case both parents were willing to participate, a preference was given to the father since fathers participate less often in research (Robinson, 2014). In total 31 parents were interviewed, including 21 mothers. The socio-demographic characteristics of the participants are shown in Table 1. Parents were asked to solely answer the questions concerning their children within the target age group if they also had children outside the target age group.

**Table 1:** Participants’ socio-demographic characteristics (*N* = 31)

	*n* (%)	*M* (SD)	Min	Max
Gender				
Men	10 (32%)			
Woman	21 (68%)			
Age		41.52 (7.02)	22	51
Education level				
Secondary school	1 (3%)			
Secondary vocational education	10 (32%)			
University of applied sciences	15 (48%)			
University	5 (16%)			
Marital status				
Married	21 (68%)			
Divorced	1 (3%)			
Living together	7 (23%)			
Single	2 (6%)			
Number of working hours p/w		26.75 (14.04)	0	50
No job	3 (10%)			
Parttime	18 (58%)			
Fulltime	9 (29%)			
Children per household		2.50 (1.26)	1	6
One child	5 (16%)			
> 1 children	26 (84%)			
Gender of the children				
Boy	29 (50%)			
Girl	29 (50%)			
Age of the children		10.17 (4.08)	3	16

*n* amount, *M* mean, *SD* standard deviation

### Data Collection

For this study, semi-structured interviews were conducted in the Netherlands by six trained interviewers. In the Netherlands, nearly all 12–25-year-olds use a smartphone daily. The weekly internet use among 8–11-year-old children has increased from ~8 h in 2007 to ~13 h in 2016, while 12–15-year-olds use the internet for over 20 h a week (Livingstone et al., 2017). With the abundance of portable devices such as smartphones, laptops, and tablets, adolescents in the Netherlands report on average 7 h and 20 min a day using digital media (Wennekers et al., 2016).

During the interview training, among others, the aim and background of the study was explained, the topic list was discussed in great detail and do’s and don’ts were handled. Using a topic list decreased the influence of the interviewers including conscious and unconscious biases (Diefenbach, 2009), and beliefs on the topic (Harry et al., 2005). On the one hand, this interview type generates more comparable data even though the interviews were conducted by more than one interviewer, but, on the other hand, gives interviewers the freedom to formulate and ask new questions based on interviewees answers, which results in richer and more detailed data (Boeije, 2010). The topic list included the following topics: 1) general background information, 2) availability of digital devices at home, 3) parents’ digital media use, 4) children’s media use, 5) positive and negative aspects of children’s media use, 6) attitudes towards children’s digital media use, 7) rules regarding digital media use and 8) differences in socialization regarding digital media use between siblings. Based on our analysis of previous knowledge and current digital developments as discussed in the introduction, as well as several socialization theories (e.g. social control theory), we hypothesized that parents must be vital in children’s digital media use and that one aspect that could play an important role is parents’ self-interest. Therefore, in this study, we were interested in the role of parents’ self-interest in children’s digital media use. Interviewers were informed about this process of thinking and were mostly interviewing parents they were acquainted with, both factors that may have influenced the research process (Holmes, 2020; Savin-Baden & Major, 2013). To get an idea of what the self-interest of parents entails and how this plays a role in dealing with their children’s digital media use, among others the following questions were asked: “To what extent do you experience positive aspects of your child’s digital media use for yourself?”, “Do you initiate or encourage digital media use in your child?”, and “Do you let your child use digital media longer than desired or agreed upon?”. After an affirmative answer, the interviewers asked for examples of specific situations and underlying reasons. Interviews took on average 35 minutes. All interviews were conducted from November 2018 to January 2019 and took place at the participants’ homes; except for three interviews that were conducted by phone. Participants signed a consent form prior to the interview, stating that they were informed about the aim of the study and able to resign at any moment. Respondent numbers were used to guarantee the anonymity of participants. The study was approved by the Ethics Review Board of the Faculty of Social and Behavioral Sciences at Utrecht University (FETC18-060).

### Data Analysis

The audio-taped interviews were transcribed intelligent verbatim, resulting in transcripts comprising an average volume of 10 pages. Subsequently, the transcripts were analyzed using NVivo 12 (Richards, 1999). Different data analysis methods were applied to answer the four research questions. First, deductive content analysis, also known as directed content analysis (Elo & Kyngäs, 2008), was used to validate our expectations about how parents deal with their children’s digital media use out of self-interest (research question 1). This method enabled us to analyze this particular aspect of the data in a detailed way. First, all text fragments that refer to parents’ self-interest in their children’s digital media use were highlighted. Next, these text fragments were coded using three predefined categories to which new categories could be added if the data gave reason to do so (Hsieh & Shannon, 2005). This allowed us to look out for the unexpected as well. The three predefined categories were derived from the topic list and included: “initiating out of self-interest”, “omitting restrictive responses out of self-interest” and “allowing out of self-interest when asked”. When it was not clear in which way parents dealt with their children’s digital media use out of self-interest, the text fragment was labeled ‘positive aspects for parents’, as these fragments provide valuable information about which different interests of parents play a role (research question 2). The category ‘having set times out of self-interest’ was added to the predefined categories, as this category emerged from the data as a fourth way in which parents’ self-interest plays a role in children’s digital media use. Since we wanted a rich description of the data and did not want our analysis to answer the second research question (about which self-interests of parents play a role in children’s digital media use) to be driven by existing knowledge, for this research question, a thematic analysis with an inductive approach at a semantic level was used (Braun & Clarke, 2006). All different forms regarding the self-interest of parents were first coded openly and then axially. Thereafter, themes have been identified that represent the different forms of parents’ self-interest in children’s digital media use by grouping codes together. The coding phase for both the deductive and inductive analysis started with two researchers independently analyzing two transcripts, after which discrepancies between coding were discussed. Two sessions of reflection on double-coding took place to reach sufficient agreement. After the coding phase, the data were summarized in tables (called the framework approach, which is commonly used for thematic analysis; Gale et al., 2013) which enabled us to compare data across as well as within participants and answer research question 3 (about the extent to which the different forms of parents’ self-interest provoke different ways of dealing with children’s media use) and 4 (comparison between parents with children of different ages). To look at differences and similarities between parents with children of different ages, the interviews were divided into parents with children in the following age categories: 3 through 5 years old (*n* = 8), 6 through 9 years old (*n* = 12), 10 through 11 years old (*n* = 9), 12 through 14 years old (*n* = 13) and 15 through 16 years old (*n* = 10). These age groups are related to the different developmental stages of children associated with differences in children’s interests, abilities, and level of autonomy (Crain, 2005).

## Results

### In Which Ways Do Parents Deal with Children’s Digital Media Use Out of Self-interest?

Almost all parents stated that they experience moments when their children’s digital media use was convenient for them. Three different ways in which these moments can be created or utilized by parents are confirmed. First, deductive analysis confirmed that parents can initiate digital media use among their children for reasons of self-interest. This means that at times children are not using digital media, parents proposed to do so. In this way, parents’ self-interest plays an active role in children’s digital media use: “Yes when I am cooking and I cannot help him and he is tired and hungry and everything fails with him then I distract him, I kind of put him on hold with a video.” (mother, son 4 years having no own devices). Second, deductive analysis confirmed that parents may omit restrictive responses to their children’s digital media use out of self-interest. In this case, parents let children use digital media longer than agreed upon or desired. In this way, parents’ self-interest plays a passive role in children’s digital media use: “Does it ever happen that you let your child use digital media longer than agreed upon or desired?” (interviewer). “Yes, it happens because one is also busy with many different other things.” (mother, son 13 years owning a smartphone, daughter 16 years owning a smartphone). Third, deductive analysis confirmed that, for reasons of self-interest, parents can allow the use of digital media when children ask for it. This is also a passive way of dealing with children’s digital media use out of self-interest: “Then it’s like ‘Can I use the PlayStation?’ and then it’s ‘Oh well, yes go ahead.’ (…) Otherwise you know how it goes: There will be a crisis and you don’t always want that.” (mother, son 8 years having an old mobile phone to use at home, daughter 16 years owning a smartphone and a laptop). Next to these three ways of dealing with children’s digital media use out of self-interest, a fourth way derived from the data: parents may set times when their children are allowed to use digital media out of self-interest. “From 5 pm, just before diner they can watch television or play a game on the iPhone.” (father, son 5 years having no own devices, son 7 years having no own devices).

### Which Self-interests of Parents Play a Role in Letting Children Use Digital Media?

Six forms of parents’ self-interest that play a role in children’s digital media use were derived from inductive analysis.

#### Being able to do other tasks without being disturbed

The most frequently mentioned self-interest of parents in letting children use digital media (71% of the parents, *n* = 22), is having free hands to do something undisturbed, such as a) household chores: “Recently, we went away for one night and I really had to pack my bag and I still had to clean the house completely, (…). At these moments, I just find it annoying when she bumps around me all the time, because then I really want to get things done in an hour. So, then I let her watch a movie on her own.” (mother, daughter 3 years having no own device), b) finishing work or having a telephone conversation: “ (…) then they don’t distract you and then you think: oh I can also make that call to a friend.” (mother, daughter 10 years owning an old mobile phone to use at home and an iPad, son 13 years owning a smartphone, iPad and Playstation). Results show that this form of self-interest can provoke all four different ways of dealing with children’s digital media.

#### Creating me-time

Me-time is time spend on one’s own to relax. Thirty-two percent of the parents (*n* = 10) mentioned that they sometimes regard their children’s digital media use as a moment to have some me-time. Examples of me-time that parents mentioned include reading a book and staying in bed a bit longer during the weekend: “Yes, when I am sitting on the couch quite comfortable or I am just reading a book. (…) Then I think, let her watch just a few more minutes, then I can relax a bit longer.” (mother, daughter 3 year having no own device). Me-time can be created by dealing with the digital media use of children in all four different ways.

#### Regulating child’s behavior

Thirty five percent of the parents (*n* = 11) indicated that digital media offer a solution in situations in which parents want the child to behave and to be calm. Situations that were mentioned are mainly in public places, such as in the town hall, restaurants, or in the company of others, but also in situations when children have to wait for a long time, for example during a long drive or during physiotherapy treatment of the parent. In these situations, digital media use is usually initiated by the parent or allowed when the child requests it: “Or sometimes we also put videos offline on Netflix so they can watch that there. Then it’s easier to have a pleasant after-dinner conversation without having your children running around in the restaurant.” (mother, son 3 years having no own device, son 5 years having no own device).

#### Avoiding a discussion with the child

Thirteen percent of the parents (*n* = 4) mentioned that they sometimes omit restrictive responses to their child’s digital media use or allow the use of digital media, because they do not want to start a discussion with their child and want to keep the atmosphere pleasant: “Does it ever happen that you allow your child to use digital media longer than actually intended?” (interviewer). “Yes. (…) Or you don’t want you to get a discussion about it again, that we will get in discussion with each other, you know, then you think’just let it go’.” (mother, son 8 years having an old mobile phone to use at home, daughter 16 years owning a smartphone and a laptop).

#### Being able to use digital medIA THEmselves

Another interest of parents that can play a role in dealing with their children’s digital media use is that parents want to use digital media themselves. Ten percent of the parents (*n* = 3) provided an example of a situation in which it appears that they omit restrictive response to their child’s digital media use for this reason: “If there is a cool football match that we all would like to see, especially the guys here at the table, then we turn a blind eye and then there is a telephone at the table (…).” (father, son 5 years having no own devices, son 7 years having no own devices).

#### Spending quality time together

From 13% of the interviews (*n* = 4), it appeared that digital media can be used to facilitate quality-time between parent and child: “We also search for lyrics very often and then the three of us are singing or listening to some songs, or we watch videos for school.” (mother, daughter 10 years having her own smartphone, daughter 14 years having her own smartphone and laptop). “You also just want to do something fun with her. And if you try to watch together, you will talk about or laugh at something fun together, then it is just fun.” (mother, daughter 3 years having no own devices). These moments are mainly created by parents proposing to use digital media together.

### Comparison between Families with Children of Different Ages

The extent to which parents’ self-interest plays a role in dealing with children’s digital media use seems to decrease as children get older. For example, all parents with children in the age category younger than 6 years old mentioned situations in which they let their children use digital media for reasons of self-interest. From the group of parents with children aged 6 to 9 years and the group of parents with children aged 10 and 11 years, the majority indicated that their self-interest is a motive for letting their children use digital media (respectively 83%, *n* = 10, and 78%, *n* = 7). For parents with children between 12 to 14 years old, this percentage is 38% (*n* = 5) and for parents with children aged 15 and 16 years this percentage is 30% (*n* = 3). Moreover, 42% of all interviewed parents (*n* = 13) indicated not letting their children use digital media to satisfy their self-interest (that often) anymore presently, but used to do this (more often) in the past: “When they were younger, you used it more often. When I wanted to have dinner, when they were little, when I was going to make diner that was the moment that they were usually the most annoying of course. And then I could not use that whining and yes, I sometimes put them in front of a children’s TV program and then yes, you benefit from it. (…) At this age, that is no longer an issue.” (mother, son 13 years owning a smartphone, daughter 16 years owning a smartphone).

The interviews also show differences between families with younger and older children in the way parents deal with their children’s digital media use out of self-interest. Parents’ self-interest mainly plays an active role in the digital media use of children under the age of 6 actively initiating digital media use or having set times for the use of digital devices. In families with children aged 6 to 11 years, we see that initiating and omitting restrictive responses to digital media use are the most common ways of how parents benefit from their children’s digital media use. The digital media use of children aged 12 to 16 years is rather passively affected by parents’ self-interest because parents omit restrictive responses to children’s digital media use when it is convenient for them. Situations in which parents benefit from the digital media use of their children by allowing it when the child asks for it are hardly mentioned by parents with children older than 11 years. Furthermore, having set times for their children to use digital media to satisfy their self-interest is not mentioned by parents with children aged 12 to 16 years old. Thus, it seems that parents mainly stimulate digital media use in younger children, while in older children parents mainly omit to limit screen time out of self-interest. In contrast to parents of younger children, almost all parents of children aged 12 to 16 years mentioned that their children have their own smartphone. This determines their access to digital devices and probably influences how parents supervise and limit their digital media use. This could explain the differences found between families with younger and older children in the way parents deal with their children’s digital media use out of self-interest.

Not only do the extent to and how parents’ self-interest plays a role in children’s digital media use differ for families with younger and older children, but also the types of self-interest play an important role. Parents of younger children allow or encourage digital media use more often to be able to do other tasks undisturbed, to have me-time, and to ensure that the child behaves and will stay calm. The interest that is mentioned most frequently by parents with older children is being busy with certain tasks. Examples of situations in which parents use digital media to ensure that children behave in public places are not mentioned in the interviews with parents with children older than 9 years. The same applies to situations in which parents let their children use digital media to have some me-time. Table 2 provides a clear overview of how parents deal with children’s digital media use out of self-interest, which type of self-interest is dealt with, and the extent to which this occurs per age category.

**Table 2:** Overview of how parents deal with children’s digital media use motivated by which self-interest and the extent to which this occurs per age category

Ways of dealing with children’s digital media use	Parents’ self-interests	3–5 years	6–9 years	10–11 years	12–14 years	15–16 years
Initiating	Being able to do other tasks	**✓**	**✓**	✓	✓	
	Having some me-time	**✓**				
	Regulating child’s behavior	**✓**	**✓**	✓	✓	
	Spending quality time together	✓	✓	✓	✓	✓
Omitting restrictive responses	Being able to do other tasks	✓	**✓**	**✓**	**✓**	✓
	Having some me-time	**✓**	✓			
	Avoiding a discussion		✓		✓	
	Being able to use digital media themselves	✓	✓		✓	✓
Permitting when asked	Being able to do other tasks	**✓**				
	Having some me-time	**✓**				
	Regulating child’s behavior	✓	✓	**✓**		
	Avoiding a discussion	✓	✓			
Having set times	Being able to do other tasks	**✓**	**✓**	✓		
	Having some me-time	**✓**	**✓**			

**✓** = revealed 4 times or more, **✓** = revealed 2*–*3 times, ✓ = revealed once

## Discussion

This qualitative study contributes to existing knowledge about the role of parents’ self-interest in the digital media use of children across different ages (3 to 16 years). In line with previous studies (Beyens & Eggermont, 2014; Hawi & Rupert, 2015; He et al., 2010), our findings show that parents regularly let children use digital media to satisfy their interests. Parents deal with children’s digital media use out of self-interest in different ways, and different forms of self-interest play a role herein. This study also shows that the role of parents’ self-interest in children’s digital media use is different for families with younger and older children.

Contributing to previous studies (e.g. Beyens & Eggermont, 2014) on the benefits parents experience by letting their children using digital media, our results show that parents’ self-interest provokes four different ways of dealing with children’s digital media use. Out of self-interest, parents can (1) initiate digital media use among their child, (2) omit restrictive responses to their child’s digital media use, (3) allow the use of digital media when their child asks for it and/or (4) have set particular times during which children are allowed to use digital media. These results show that, motivated by self-interest, parents can utilize, extend or create the moments during which children are using digital media, which can result in children using digital media more often and/or longer than desirable.

Parents’ self-interest in children’s digital media use may include (1) being able to do other tasks undisturbed, (2) having some me-time, (3) ensuring that the child behaves, (4) avoiding a discussion with the child, (5) being able to use digital media themselves, and (6) spending quality time together. With the exception of being able to use digital media themselves, these findings are in line with findings from previous research into the benefits of children’s digital media use for parents (Evans et al., 2011; Götz et al., 2007; He et al., 2005; Hesketh et al., 2012). To our knowledge, the use of digital media by parents themselves is a perceived benefit not shown in previous studies. This may be explained by the current era compared to the period in which the previous studies were conducted. Digital media are a greater part of everyday life today than a decade ago (Cloïn et al., 2013). The self-interest that has been mentioned the most by far is being able to do other tasks undisturbed. Several studies have shown that this benefit associated with children’s digital media use can make it difficult for parents to limit children’s screen time (Evans et al., 2011; Götz et al., 2007; He et al., 2005; Hesketh et al., 2012).

The current study also demonstrates how parents deal with children’s digital media use out of self-interest differs per type of self-interest. Having a moment to do other tasks undisturbed and having me-time can be obtained in all four different ways. However, if parents want to ensure that children behave and stay calm, digital media use is primarily initiated or permitted after being asked by the child. To be able to use digital media themselves, parents omit restrictive responses to children’s digital media use. Last, to create quality time, digital media use is initiated by parents. Interventions aimed at reducing children’s screen time can make use of this knowledge, since this knowledge may help parents to become aware of how their self-interest plays a role in dealing with their children’s digital media use and help them to recognize such situations more easily.

The extent to which and how parents’ self-interest plays a role in children’s digital media use differs between families with younger children and older children. The interviews illustrate that parents with younger children provide digital media actively to the child or create set times at which the child is allowed to use digital media, so they do not have to entertain the child by themselves. Parents indicate that they mainly do this to be free to do other tasks, to have some time for themselves, or to ensure that the child behaves. Because of the mobility of smartphones and tablets, they are not only used at home but also in public places (e.g. restaurants) or on the road to regulate children’s behavior. Researchers have notified that this might result in children with insufficiently developed abilities to regulate their behavior, for example during boredom (Radesky et al., 2016). Furthermore, the finding that parents with younger children let their children use digital media so that they can do other tasks, implies that children use digital media independently already from a young age instead of together with a parent. This increases the chance of children getting in touch with online content that is inappropriate for their age (Paavonen et al., 2009).

Parents’ self-interest seems to play less of a role in the digital media use of children aged 12 and older. This may be because older children, compared to younger children, are better and longer able to enjoy themselves independently, whereby parents have to let their children use digital media less to satisfy their self-interest. Interestingly, adolescents enjoy themselves often with digital media. The use of digital media is more normative among older children (Boeke et al., 2017), and will therefore be more tolerated by parents. When the digital media use of older children is influenced by parents’ self-interest, this mainly occurs passively: parents omit restrictive responses when their children’s media use is convenient for them. Situations in which digital media use is permitted when asked for and having set times at which digital media can be used for parents’ own interests are (hardly) ever mentioned in the interviews with parents of older children (>12 years). Our finding that most children of this age have at least their own smartphone might explain these findings as this enlarges the access to digital devices and the subsequent online world. They probably do not need to ask permission for the use of it. Furthermore, adolescents aged 12 and older generally use digital media throughout the whole day (Wennekers et al., 2016), resulting in parents of older children often trying to restrict digital media by setting agreements about when they are not allowed to use digital media, instead of when they are allowed to use, as is often the case with younger children.

### Limitations

To our knowledge, this is the first study that provides more insights into how parents’ self-interest plays a role in the digital media use of toddlers and preschoolers as well as elementary and secondary school children, and which types of self-interests play a role herein. However, some limitations should be mentioned. First, although targeted sampling has led to a diverse sample (in terms of parent’s and children’s gender and age, level of education, living in a city or urban area), single-parent and one-child families are underrepresented. In these families, parents’ self-interest is expected to play an even bigger role in dealing with children’s digital media use, because single parents cannot divide the household and upbringing tasks with the other parent. Thus, digital media might offer a solution more often and in more different situations. The same applies to families with one child who does not have any brothers or sisters with whom they can play or spend time with. Second, two different interview modes were used; nearly all interviews were held in person, yet three were conducted by phone. Telephone interviews may be better to discuss sensitive topics and may allow participants to feel more relaxed (Novick, 2008). However, face-to-face interviews may provide more rich data as visual aspects such as facial and body expression can be taken into account. Yet, there is little evidence that the quality of findings is compromised when interview data is collected by phone (e.g., Azis & Kenford, 2004; Novick, 2008; Sturges & Hanrahan, 2004). Third, some of the participants were acquaintances of the interviewers. This might have influenced participant’s answers. Although we formulated the interview questions as neutral as possible, due to the sensitivity of the topic, participants, in general, may have provided socially desirable answers during the interview. As a result, parents’ self-interest may play a more important role in children’s digital use of media than the interviews reveal. This may be even more true for parents of older children because the digital media use of these children is more normative, and parents may justify their tolerant attitude and behavior towards digital media use as a form of ‘autonomy granting’. ‘Autonomy granting’ means that children get increasingly more freedom from their parents to make their own choices as they are getting older (Supple et al., 2009). In this way adolescents learn to take responsibility for their behavior. Parents may indicate that they do not intervene in their child’s digital media use because they want to give their child more autonomy, while possibly parents’ self-interest also plays a role here. Future longitudinal studies are needed to investigate how parents’ self-interest plays a role in children’s level of digital media use.

## Conclusion and Implications

This qualitative study provides a better insight into the self-interest of parents in relation to the digital media use of children from 3 to 16 years old. The interviews show that letting children use digital media can fulfill certain needs of parents and that this can result in children using digital media more often and/or longer. Especially in families with younger children, parents’ self-interest seems to play a significant role. These parents mainly use digital media out of self-interest by initiating digital media with the child, or by managing set times at which the child can use digital media. When parents of older children provide digital media for their self-interest, this is generally done more passively by not intervening during digital media use. For parents with both younger and older children, the possibility to do other tasks undisturbed seems to be the most important reason to let their children use digital media. Additionally, for parents of younger children, creating me-time and keeping the child calm and behaving are two additional motives to provide digital media. Interventions aimed at reducing children’s screen time could utilize this knowledge by taking into account these parental interests. This is especially important for interventions that focus on parents of young children. These parents in particular should be offered concrete and feasible alternatives when it comes to activities that children can enjoy independently (Carson et al., 2014). Furthermore, interventions could focus on enhancing parenting skills by providing them with other tools to regulate their children’s behavior instead of digital media (He et al., 2005). Thus, the establishment of unhealthy media habits at a young age, which may continue in later life, can be prevented (Carson et al., 2014).
